# Significant association of *SREBP-2 *genetic polymorphisms with avascular necrosis in the Korean population

**DOI:** 10.1186/1471-2350-9-94

**Published:** 2008-10-27

**Authors:** Tae-Ho Kim, Jeong-In Baek, Jung Min Hong, Su-Jin Choi, Hye-Jin Lee, Hyun-Ju Cho, Eui Kyun Park, Un-Kyung Kim, Shin-Yoon Kim

**Affiliations:** 1 Skeletal Diseases Genome Research Center, Kyungpook National University Hospital, 44-2 Samduk 2-ga, Jung-gu, Daegu, 700-412, Republic of Korea; 2 Department of Biology, College of Natural Sciences, Kyungpook National University, Daegu, 702-701, Republic of Korea; 3 Department of Medicine, Kyungpook National University School of Medicine, 101 Dongin-dong 2 Ga, Jung-gu, Daegu, 700-422, Republic of Korea; 4 Department of Pathology and Regenerative Medicine, School of Dentistry, Kyungpook National University, 188-1 Samduk 2-ga, Jung-gu, Daegu, 700-412, Republic of Korea; 5 Department of Orthopedic Surgery, Kyungpook National University School of Medicine, 101 Dongin-dong 2 Ga, Jung-gu, Daegu, 700-422, Republic of Korea

## Abstract

**Background:**

It is known that steroid usage and alcohol abuse are major etiological factors in the development of avascular necrosis (AVN), a bone disease that produces osteonecrosis of the femoral head. The facilitation of fat biosynthesis by steroids and alcohol disrupts the blood supply into the femoral head. *SREBP-2 *plays a central role in the maintenance of lipid homeostasis through stimulating expression of genes associated with cholesterol biosynthetic pathways. The aim of this study was to examine the association between the polymorphisms of the *SREBP-2 *gene and AVN susceptibility in the Korean population.

**Methods:**

Four single nucleotide polymorphisms (SNP) in the *SREBP-2 *gene, IVS1+8408 T>C (rs2267439), IVS3-342 G>T (rs2269657), IVS11+414 G>A (rs1052717) and IVS12-1667 G>A (rs2267443), were selected from public databases and genotyped in 443 AVN patients and 273 control subjects by using single-based extension (SBE) genotyping.

**Results:**

The minor allele (C) frequency of rs2267439 showed a significant protective effect on AVN (P = 0.01, OR; 0.75, 95% CI; 0.604–0.935), and the genotype frequencies of this polymorphism were also different from the controls in all alternative analysis models (P range, 0.009–0.03, OR; 0.647–0.744). In contrast, rs1052717 and rs2267443 polymorphisms were significantly associated with AVN risk. Further analysis based on pathological etiology showed that the genotypes of rs2267439, rs1052717 and rs2267443 were also significantly associated with AVN susceptibility in each subgroup.

**Conclusion:**

This study is the first report to evaluate the association between *SREBP-2 *gene polymorphisms and the susceptibility of AVN in the Korean population.

## Background

Avascular necrosis (AVN), also known as osteonecrosis of the femoral head (ONFH), is a devastating bone disease that develops symptoms of articular destruction and bone collapse of the femoral head due to a disturbance in the supply of blood [1]. This disease mainly occurs in middle aged men, between 30 and 50 years of age. The pathogenic factor of AVN is not definite, but many previous studies have suggested that long term steroid usage [1-5] and alcohol abuse [6,7] are associated with AVN, and in some case it can also be idiopathic. These factors have a harmful influence on oxygen and nutrient supply to the bone through blood vessels in direct or indirect pathways. Steroid administration and alcohol abuse induce an increase of fatty vesicles in the circulation of the blood, causing fat embolism and increased lipid precipitation in osteocytes within the femoral head. Some previous experiments using animal models verified that alcohol facilitates the overgrowth of fat cells and inhibits osteogenesis in the femoral head [8]. As a result, these changes obstruct the flow of blood and induce intraosseous hypertension, thus leading to bone collapse.

Sterol regulatory element binding transcription proteins (SREBPs) which belong to the basic helix-loop-helix family of transcription factors have been associated with lipogenesis, adipocyte development and cholesterol homeostasis [9]. Three members of the SREBP family, *SREBP-1a*, *SREBP-1c *and *SREBP-2*, have been identified to date [10]. *SREBP-1a *contributes to cholesterol and fatty acid metabolisms, and *SREBP-1c *regulates expression of certain genes associated with biosynthesis of fatty acid [11]. *SREBP-2*, which is encoded by a separate gene on human chromosome 22, plays a central role in the maintenance of lipid homeostasis through stimulating expression of genes associated with the cholesterol biosynthetic pathways [12]. *SREBP-2 *exists in the membrane of endoplasmic reticulum forming a complex with SREBP cleavage activating protein (SCAP) [13]. When cholesterol is deficient in cells, SCAPs transport *SREBP-2 *from endoplasmic reticulum to the Golgi complex, where the proteins are cleaved by two protease known as Site-1 protease (S1P) and Site-2 protein (S2P) and are maturated to a soluble transcription factor to stimulate the expression of the target genes. The target genes of maturated *SREBP-2 *encode enzymes with important roles in the synthesis and uptake pathway of cholesterol and triglyceride [14].

In this study, we hypothesized that *SREBP-2 *activity is responsible for the development of AVN and that the variants of *SREBP-2 *gene are associated with the susceptibility to AVN. To test this hypothesis, four *SREBP-2 *single nucleotide polymorphisms (SNPs) which show high heterozygosities in Asian populations, especially Han Chinese and Japanese, were selected from the public database, and their association with susceptibility to AVN in the Korean population was evaluated.

## Methods

### Subjects

Blood samples and information were obtained from 443 unrelated patients with AVN (366 men, 77 women; age: 49.7 ± 13.3) and 273 control subjects (206 men, 67 women; age: 52.1 ± 10.6) visiting Kyungpook National University Hospital (Daegu, Korea) between 2002 and 2006. Diagnosis was established by the evidence of symptomatic AVN using anteroposterior, lateral-pelvic radiographs and magnetic resonance imaging (MRI) in stage 1 of association research circulation osseous (ARCO) classification system, and by plain radiographs in stages 2, 3 and 4. Patients with a demonstrable history of direct trauma or with a possible combination of causes were excluded. Based on etiological factors, the patients were subgrouped into idiopathic (181 cases), alcohol-induced (206 cases) and steroid-induced (56 cases) groups. Steroid-induced AVN was defined by a history of taking 1800 mg prednisolone or an equivalent over 4 weeks with nephritic syndrome, systemic lupus erythematosus, rheumatoid arthritis, allergic asthma, or organ transplantation [15]. Alcohol-induced AVN was defined by the consumption of more than 400 ml of pure ethanol per week, or by the observation of alcohol induced fatty liver and liver cirrhosis. Control subjects were recruited from spouses of the patients and the general population and were considered normal if they had no hip pain and if anteroposterior and frog-leg lateral pelvic radiographs did not reveal any lesions with sclerotic margins or subchondral collapse consistent with AVN. All individuals provided informed consent for their participation in the study, and this project was approved by our Institutional Review Board.

### Polymerase chain reaction (PCR) amplification

Genomic DNA was isolated from peripheral blood leukocytes using a FlexiGene DNA Kit (QIAGEN, Valencia, CA, USA). All PCR reactions were performed in a 25 μl volume containing 1× PCR buffer (Solgent, Korea), 10 mM deoxynucleotide triphosphate (dNTP), 10 pmol each of forward and reverse oligonucleotide primer, 1 U/μl Taq DNA polymerase (Solgent, Korea), and 25 ng genomic DNA. The parameters for PCR consisted of a denaturation cycle at 95°C for 15 min, followed by 35 cycles at 95°C for 20 sec, 55–57°C for 40 sec, 72°C for 1 min, a final extension cycle at 72°C for 5 min, and a stabilization step at 4°C. PCR was performed using a PTC-200 thermal cycler and an iCycler™ Thermal cycler (BIO-RAD). For purification of the PCR products, an ExoSAP IT Kit (USB Corp., Cleveland, OH, USA) was used.

### Genotype analysis

Genotyping was performed by the single-based extension (SBE) method with a SNaPshot Kit (Applied Biosystems Corp., Foster City, CA, USA). The SBE reaction mixture was prepared according to the manufacturer's instructions. The primer extension reaction was performed in 25 cycles at 96°C for 10 sec, 50°C for 5 sec, and 60°C for 30 sec. To resolve the excess primers, 1 unit of shrimp alkaline phosphatase (USB Corp., Cleveland, OH, USA) was added to the reaction mixture, and the mixture was incubated at 37°C for 60 min, followed by 15 min at 80°C for enzyme inactivation. The loading solution containing an injection marker was added to inactivate the SNaPshot reaction mixtures according to the recommendations of the manufacturer. Genotype scanning was performed using an ABI 3130 × l genetic analyzer, and the resulting files were analyzed by using Gene Mapper software program (Applied Biosystems Corp., Foster City, CA, USA).

### Statistical analysis

The Hardy-Weinberg equilibrium (HWE) was tested to determine significant deviation of the genotype frequency from each single nucleotide polymorphism (SNP) by using the χ^2 ^test. Logistical regression analyses were used to calculate the odds ratios (ORs), 95% confidence intervals (CIs) and haplotypes, controlling for sex and age as covariates, with three alternative models (codominant, dominant and recessive). The linkage disequilibrium (LD) between loci was measured using the absolute value of Lewontin's D' (|D'|) and *r*^2 ^[16]. Haplotypes of the *SREBP-2 *gene were analyzed using Haploview version 3.32 based on the expectation maximization (EM) algorithm [17]. All analyses were two-tailed, and a P value < 0.05 was considered statistically significant. Statistics were performed using SAS 9.1 (SAS Institute Inc., Cary, NC, USA).

## Results

**Figure 1:**
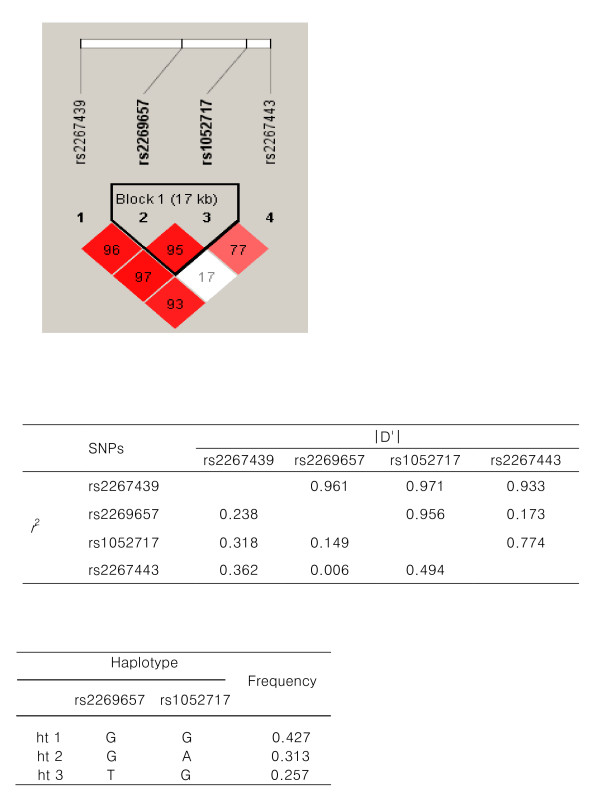
**Linkage disequilibrium coefficients and haplotypes of *SREBP-2 *polymorphisms **A. **Linkage disequilibrium (LD) among *SREBP-2 *polymorphisms.B. **LD coefficients (| D'| and *r*^2^) between *SREBP-2 *polymorphisms. **C. **Haplotypes and their frequencies between two linked SNPs, rs2269657 and rs1052717, in a LD block.

To investigate the association of *SREBP-2 *gene polymorphisms with AVN, we selected four intronic SNPs, rs2267439, rs2269657, rs1052717 and rs2267443, from public databases by considering their allele frequencies and positions, and analyzed these polymorphisms in 443 AVN patients and 273 control subjects. LD coefficients (|D'|) and *r*^2 ^were calculated, and rs2269657 and rs1052717 demonstrated tight LD (Fig. 1A and 1B). The haplotypes and their frequencies in the LD block between rs2269657 and rs1052717 are showed in Figure 1C.

The genotype frequencies of all the polymorphisms were in accordance with Hardy-Weinberg equilibrium in both cases and control groups (Table 1). The P values of each polymorphism were analyzed by logistic analysis with respect to a comparison between AVN patients and the controls. As shown in Table 2, the allele and genotype frequencies of rs2267439 in patients were significantly different from those in the controls. The minor allele (C) frequency of rs2267439 showed a significant protective effect on AVN (P = 0.01, OR; 0.75, 95% CI; 0.604–0.935), and the genotype frequencies of this polymorphism were also different from the controls in all alternative analysis models (P range; 0.009 – 0.03, OR; 0.647–0.744). In contrast to the characteristics of polymorphism rs2267439, rs1052717 and rs2267443 polymorphisms were significantly associated with the risk of AVN. The AA genotype frequency in rs1052717 was higher in patients when calculated using the recessive model (P = 0.04, OR; 1.8, 95% CI; 1.034–3.120). The rs2267443 allele and genotype frequencies showed a significantly higher risk factor in AVN patients compared with those in controls with P values 0.04 (OR; 1.27, 95% CI; 1.006–1.590) and 0.04 (OR; 1.26, 95% CI; 1.003–1.579) in the codominant model. Though the P value of rs2267443 genotype frequency in the recessive model was slightly greater than 0.05, it showed the tendency of weak association with the risk of AVN development. No differences in the frequencies of the allele and genotypes were seen in patients and the controls in the case rs2269657 polymorphisms.

**Table 1:** Frequencies of SREBP-2 polymorphisms in AVN and normal subjects

Loci	Position	rs#	Genotype					MAF	Heterozygosity	HWE				
										
										Control	Case all	Alcohol	Idiopathic	Steroid
IVS1+8408 T>C	Intron1	rs2267439		TT	CT	CC	N			0.714	0.619	0.653	1	0.428
			AVN	159	218	66	443	0.395	0.492					
			CTL	77	138	55	270	0.459	0.511					
IVS3-342 G>T	Intron2	rs2269657		GG	GT	TT	N			0.637	0.71	0.469	1	1
			AVN	241	149	32	422	0.263	0.353					
			CTL	146	107	16	269	0.258	0.398					
IVS11+414 G>A	Intron11	rs1052717		GG	AG	AA	N			0.301	0.331	0.334	0.184	0.237
			AVN	202	186	53	441	0.331	0.422					
			CTL	132	120	19	271	0.292	0.443					
IVS12-1667 G>A	Intron11	rs2267443		GG	AG	AA	N			0.891	0.267	1	0.077	1
			AVN	169	192	69	430	0.384	0.447					
			CTL	116	121	29	266	0.337	0.455					

**Table 2:** Association of SREBP-2 gene polymorphisms between the AVN patients and controls

Loci	Sub-group	Genotype				Allele 2 vs. 1		Codominant Heterozy		Dominant		Recessive	
											
		11	12	22	MAF			(11 vs 12 vs 22)*		12 + 22 vs. 11		22 vs. 11 + 12	
						
						OR (95% CI)	P†	OR (95% CI)	P†	OR (95% CI)	P†	OR (95% CI)	P†
IVS1+8408 T>C		TT	CT	CC									
											
(rs2267439)	CTL	77	138	55	0.459								
	AVN	159	218	66	0.395	**0.751** **(0.604–0.935)**	**0.010**	**0.744** **(0.595–0.930)**	**0.009**	**0.702** **(0.504–0.978)**	**0.036**	**0.647** **(0.433–0.964)**	**0.033**
IVS3-342 G>T		GG	GT	TT									
											
(rs2269657)	CTL	146	107	16	0.258								
	AVN	241	149	32	0.263	1.069 (0.835–1.369)	0.596	1.070 (0.834–1.372)	0.595	1.039 (0.762–1.415)	0.810	1.305 (0.697–2.442)	0.406
IVS11+414 G>A		GG	AG	AA									
											
(rs1052717)	CTL	132	120	19	0.292								
	AVN	202	186	53	0.331	1.207 (0.954–1.525)	0.117	1.206 (0.954–1.525)	0.118	1.133 (0.835–1.539)	0.423	**1.796** **(1.034–3.120)**	**0.038**
IVS12-1667 G>A		GG	AG	AA									
											
(rs2267443)	CTL	116	121	29	0.337								
	AVN	169	192	69	0.384	**1.265** **(1.006–1.590)**	**0.044**	**1.259** **(1.003–1.579)**	**0.047**	1.254 (0.916–1.718)	0.157	1.589 (0.996–2.535)	0.052

*11: homozygotes for the major allele, 12: heterozygotes and 22: homozygotes for the minor allele.

† Logistic regression analyses were used for calculating. bold: P value < 0.05

Further analysis based on pathological etiologies such as idiopathic, alcohol-induced or steroid-induced showed that the allele and genotype frequencies of rs2267439 in the idiopathic subgroup were significantly different than those in the controls, suggesting that this polymorphism had a protective effect on AVN development (Table 3). However, rs1052717 and rs2267443 polymorphisms were significantly associated with increased risk of AVN in the idiopathic and alcohol subgroup, respectively.

**Table 3:** Association of SREBP-2 gene polymorphisms between the AVN subgroup patients and controls

Loci	Sub-group	Genotype				Allele 2 vs. 1		Codominant Heterozy		Dominant		Recessive	
											
		11	12	22	MAF			(11 vs 12 vs 22)*		12 + 22 vs. 11		22 vs. 11 + 12	
						
						OR (95% CI)	P†	OR (95% CI)	P†	OR (95% CI)	P†	OR (95% CI)	P†
		TT	CT	CC									
											
IVS1+8408 T>C	Alc	74	102	30	0.393	0.764 (0.581–1.004)	0.054	0.759 (0.575–1.002)	0.052	0.744 (0.494–1.121)	0.157	0.629 (0.379–1.045)	0.073
(rs2267439)	Idio	69	85	27	0.384	**0.701** **(0.532–0.923)**	**0.011**	**0.695** **(0.525–0.920)**	**0.011**	**0.625** **(0.417–0.937)**	**0.023**	0.617 (0.368–1.034)	0.067
	Ster	16	31	9	0.438	0.865 (0.560–1.335)	0.511	0.860 (0.553–1.339)	0.504	0.940 (0.479–1.844)	0.857	0.677 (0.301–1.523)	0.346
		GG	GT	TT									
											
IVS3-342 G>T	Alc	115	74	16	0.259	1.017 (0.747–1.386)	0.914	1.016 (0.751–1.375)	0.916	1.021 (0.694–1.501)	0.917	1.022 (0.495–2.109)	0.954
(rs2269657)	Idio	94	73	14	0.279	1.159 (0.855–1.570)	0.342	1.165 (0.855–1.589)	0.333	1.153 (0.785–1.695)	0.468	1.431 (0.675–3.030)	0.350
	Ster	32	20	2	0.222	0.951 (0.568–1.592)	0.848	0.949 (0.561–1.604)	0.845	0.989 (0.527–1.858)	0.973	0.704 (0.150–3.297)	0.656
		GG	AG	AA									
											
IVS11+414 G>A	Alc	95	84	25	0.328	1.241 (0.925–1.665)	0.150	1.244 (0.925–1.672)	0.149	1.143 (0.779–1.678)	0.493	**2.037** **(1.028–4.034)**	**0.041**
(rs1052717)	Idio	85	72	24	0.332	1.214 (0.910–1.621)	0.188	1.214 (0.909–1.621)	0.189	1.092 (0.747–1.598)	0.649	**1.998** **(1.055–3.783)**	**0.034**
	Ster	22	30	4	0.339	1.230 (0.778–1.945)	0.376	1.258 (0.775–2.042)	0.352	1.452 (0.782–2.695)	0.237	0.966 (0.295–3.163)	0.954
											
		GG	AG	AA									
IVS12-1667 G>A	Alc	74	95	30	0.389	**1.380** **(1.036–1.837)**	**0.027**	**1.382** **(1.036–1.844)**	**0.028**	**1.495** **(1.007–2.220)**	**0.046**	1.575 (0.877–2.828)	0.128
(rs2267443)	Idio	73	71	31	0.380	1.236 (0.930–1.642)	0.144	1.226 (0.927–1.620)	0.153	1.126 (0.759–1.669)	0.556	**1.763** **(1.015–3.059)**	**0.044**
	Ster	22	26	8	0.375	1.334 0.849–2.094)	0.211	1.340 (0.849–2.114)	0.209	1.408 (0.749–2.647)	0.288	0.566 (0.637–3.852)	0.328

*11: homozygotes for the major allele, 12: heterozygotes and 22: homozygotes for the minor allele.

† Logistic regression analyses were used for calculating. bold: P value < 0.05

Based on LD coefficients, haplotype frequencies were compared between the patients and the controls. As shown in Table 4, the GG haplotype frequencies in patients were significantly lower than those in the controls in the dominant and codominant models (P range; 0.02–0.03, OR; 0.67–0.78). In contrast, the GA haplotype demonstrated a risk factor to AVN in a recessive model (P value; 0.008, OR; 2.24, 95% CI; 1.231–4.069).

**Table 4:** Association of SREBP-2 haplotypes between the AVN patients and controls

Haplotype	Genotype	Controls (%)	Patients (%)	Codominant		Dominant		Recessive	
				OR (95% CI)	P*	OR (95% CI)	P*	OR (95% CI)	P*

ht1	ht1/ht1	51 (19.03)	73 (16.67)	**0.78** **(0.624–0.975)**	**0.029**	**0.672** **(0.481–0.94)**	**0.020**	0.799 (0.536–1.192)	0.272
(G-G)	ht1/-	143 (53.36)	209 (47.72)						
	-/-	74 (27.61)	156 (35.62)						
ht2	h2/ht2	15 (5.6)	53 (12.1)	1.245 (0.981–1.581)	0.071	1.128 (0.83–1.534)	0.440	**2.238** **(1.231–4.069)**	**0.008**
(G-A)	ht2/-	122 (45.52) 131 (48.88)	184 (42.01) 201 (45.89)						
ht3	ht3/ht3	16 (5.97)	31 (7.08)	1.081 (0.842–1.388)	0.542	1.068 (0.783–1.456)	0.678	1.245 (0.663–2.336)	0.495
(T-G)	ht3/-	105 (39.18)	168 (38.36)						
	-/-	147 (54.85)	239 (54.57)						

Haplotypes; rs2269657, rs1052717

*Logistic regression analyses were used for calculating. bold: P value < 0.05

## Discussion

Numerous studies about bone necrosis have suggested that many factors, such as steroid usage, alcoholism, infections, coagulation defects, and some autoimmune diseases are closely related with AVN susceptibility. However, etiological and pathological mechanisms of AVN have not yet been thoroughly investigated. In the pathogenic mechanisms suggested to date, a vascular hypothesis is considered to be most persuasive. It assumes that the combined effect of many metabolic factors that induce the interception of blood flow in the femoral head raises intraosseous pressure leading to osteonecrosis and eventual bone collapse [18-21]. In particular, steroid usage and alcoholism increase the fat volume in bone marrow and blood, causing the interruption of blood flow and deposition of fat in the femoral head. Numerous studies have identified that hyperlipidemia in the femoral head induced by steroid and alcohol use is associated with AVN [22-26]. However, the fact that the frequency of AVN occurrence is not always in proportion to the period and concentration of steroid use and alcohol intake indicates that individual disparity of susceptibility for these factors is an important element of the disease [27].

The *SREBP-2 *gene encodes the membrane-bound transcription factor controlling the expression of genes for the regulation of cholesterol homeostasis [12]. Its protein product, SREBP-2, regulates several genes for sterol homeostasis by recognizing the cholesterol levels in the cell, and generally producing negative feedback [28]. Considering the importance of *SREBP-2 *in the regulation of lipid metabolism, it is more likely that AVN susceptibility may be associated with *SREBP-2 *polymorphisms.

The results of our study provide evidence of an association between *SREBP-2 *gene polymorphisms and AVN. One polymorphism was found to be protective, and two were found to increase the risk of the disease (Table 2). Moreover, the protective effect of rs2267439 was associated with the idiopathic subgroup, which was in contrast to risk factor rs1052717 and rs2267443 in the alcohol-induced and idiopathic groups, respectively. Mont et al. (1995) reported that most idiopathic AVN originates from increased alcohol consumption, and Wang et al (2003) showed alcohol-induced adipogenesis as a model for the development of osteonecrosis, especially in patients with long-term and excessive use of alcohol [8,29]. In addition, haplotype GA showed as a risk factor for AVN, but AVN risk for haplotype GG in the patient group was significantly lower than in the control group. This result suggests that the rs2269657 G allele might provide a protective effect against AVN as a result of interaction with rs1052717, even though rs2269657 alone was not associated with the disorder. Along with genetic factors, chronic alcohol consumption may promote the malfunction of *SREBP-2 *which is directly or indirectly related to the development of AVN. Therefore, our study suggests that *SREBP-2 *gene polymorphisms may be one of the most important genetic factors in AVN susceptibility in the Korean population. To further substantiate this hypothesis, functional studies of *SREBP-2 *ethanol regulation are required, and the polymorphisms analyzed in this study may contribute to further studies regarding *SREBP-2 *function and AVN development.

## Conclusion

In summary, this study is the first report showing an association of *SREBP-2 *polymorphisms with a susceptibility to AVN. Future functional studies are needed to demonstrate that variations of *SREBP-2 *contribute to the development of AVN.

## Competing interests

The authors declare that they have no competing interests.

## Authors' contributions

TH and JI performed genome sequence analysis, produced the results, and drafted the manuscript. JI, JM, SJ, HJ, and HJ contributed to the preparation of samples and provided technical assistance. EK, SY and UK contributed to the conception of the study, participated in the interpretation of the results, and supervised the study. All authors read and approved the final manuscript.

## Pre-publication history

The pre-publication history for this paper can be accessed here:


